# Engineering the yeast *Yarrowia lipolytica* for the production of therapeutic proteins homogeneously glycosylated with Man_8_GlcNAc_2_ and Man_5_GlcNAc_2_

**DOI:** 10.1186/1475-2859-11-53

**Published:** 2012-05-01

**Authors:** Karen De Pourcq, Wouter Vervecken, Isabelle Dewerte, Albena Valevska, Annelies Van Hecke, Nico Callewaert

**Affiliations:** 1 Unit for Medical Biotechnology, Department for Molecular Biomedical Research, VIB, Technologiepark 927, B-9052, Ghent, Belgium; 2 Department of Biochemistry and Microbiology, Ghent University, K.L.-Ledeganckstraat 35, B-9000, Ghent, Belgium; 3 Department of Biomedical Molecular Biology, Ghent University, Technologiepark 927, B-9052, Ghent, Belgium; 4 Oxyrane Belgium, Technologiepark 3, B-9052, Ghent, Belgium

## Abstract

**Background:**

Protein-based therapeutics represent the fastest growing class of compounds in the pharmaceutical industry. This has created an increasing demand for powerful expression systems. Yeast systems are widely used, convenient and cost-effective. *Yarrowia lipolytica* is a suitable host that is generally regarded as safe (GRAS). Yeasts, however, modify their glycoproteins with heterogeneous glycans containing mainly mannoses, which complicates downstream processing and often interferes with protein function in man. Our aim was to glyco-engineer *Y. lipolytica* to abolish the heterogeneous, yeast-specific glycosylation and to obtain homogeneous human high-mannose type glycosylation.

**Results:**

We engineered *Y. lipolytica* to produce homogeneous human-type terminal-mannose glycosylated proteins, *i.e.* glycosylated with Man_8_GlcNAc_2_ or Man_5_GlcNAc_2_. First, we inactivated the yeast-specific Golgi α-1,6-mannosyltransferases *Yl*Och1p and *Yl*Mnn9p; the former inactivation yielded a strain producing homogeneous Man_8_GlcNAc_2_ glycoproteins. We tested this strain by expressing glucocerebrosidase and found that the hypermannosylation-related heterogeneity was eliminated. Furthermore, detailed analysis of N-glycans showed that *Yl*Och1p and *Yl*Mnn9p, despite some initial uncertainty about their function, are most likely the α-1,6-mannosyltransferases responsible for the addition of the first and second mannose residue, respectively, to the glycan backbone. Second, introduction of an ER-retained α-1,2-mannosidase yielded a strain producing proteins homogeneously glycosylated with Man_5_GlcNAc_2_. The use of the endogenous LIP2pre signal sequence and codon optimization greatly improved the efficiency of this enzyme.

**Conclusions:**

We generated a *Y. lipolytica* expression platform for the production of heterologous glycoproteins that are homogenously glycosylated with either Man_8_GlcNAc_2_ or Man_5_GlcNAc_2_ N-glycans. This platform expands the utility of *Y. lipolytica* as a heterologous expression host and makes it possible to produce glycoproteins with homogeneously glycosylated N-glycans of the human high-mannose-type, which greatly broadens the application scope of these glycoproteins.

## Background

The production of biopharmaceuticals, which are increasingly dominating the pharmaceutical industry, requires powerful heterologous expression systems. Lately, yeast expression hosts have attracted much interest for several reasons. They are easy to handle, they grow rapidly on simple, chemically defined media, they are cost effective, there is no risk of contamination with infectious agents originating from animal products, and they can efficiently secrete recombinant proteins, which simplifies downstream processing. But one of the most important features of yeast expression systems is their ability to perform eukaryotic post-translational protein modifications, such as N- and O-glycosylation, disulfide bond formation, and oligomerization, which are often crucial for the functionality of therapeutic glycoproteins.

**Figure 1:**
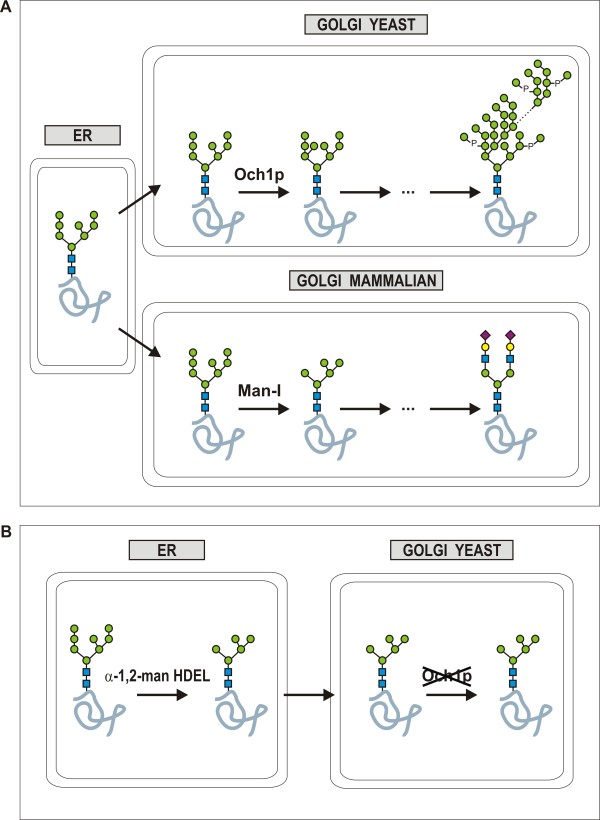
**N-glycosylation in wild type strains and the approach used to engineer the yeast pathway.** (**A**) Standard N-glycosylation in yeast and mammalian cells. The early N-glycan steps in the ER are strongly conserved between higher and lower eukaryotes until the stage at which folded proteins bearing the eukaryotic common high mannose Man_8_GlcNAc_2_ glycan structure (isomer B) leave the ER and enter the Golgi apparatus. At this point, the glycans undergo further species and cell-type specific processing. The pathways in the Golgi complex diverge notably. In higher eukaryotes, the Man_8_GlcNAc_2_ structure is further trimmed to Man_5_GlcNAc_2_ by mannosidase I and can then be further modified to complex glycans (bottom). In yeast, the Man_8_GlcNAc_2_ glycan structure is further elongated with mannoses by mannosyl- and phosphomannosyltransferases, and in some cases this results in hypermannosylation (top). (**B**) Glyco-engineering in yeast. A knock-out of the *OCH1* gene prevents the elongation of Man_8_GlcNAc_2_ glycans (isomer B), and upon expression of the HDEL-tagged α-1,2-mannosidase, Man_5_GlcNAc_2_ glycans are formed. Conforming to the representation proposed by the Consortium for Functional Glycomics Nomenclature Committee, the green and yellow spheres represent mannose (Man) and galactose (Gal), respectively, blue squares represent N-acetylglucosamine (GlcNAc) residues and the red diamonds represent sialic acid (Sia).

However, mammalian cells and yeast cells share only the initial stages of the N-glycosylation pathway, and so their glycosylation patterns are very different. Yeasts modify proteins with heterogeneous high-mannose glycan structures by the action of yeast-specific Golgi mannosyltransferases. In some cases, this leads to hypermannosylation (Figure 1A, top). Hypermannosylation in yeasts increases heterogeneity, can hamper downstream processing, and can even lead to an immunogenic response in humans. On the contrary, in mammalian cells, high-mannose N-glycans exiting the endoplasmic reticulum (ER) are trimmed to Man_5_GlcNAc_2_ in the Golgi and are usually further modified into complex-type glycans (Figure 1A, bottom). To adapt yeast for the production of biopharmaceuticals, the endogenous yeast glycosylation pathway can be engineered to produce glycoproteins with homogeneous human high-mannose type glycans (Figure 1B).

Terminal mannosylation of glycoproteins is useful for several applications. For example, successful antibody-directed enzyme pro-drug therapy (ADEPT) requires rapid tissue clearance of the drug. For instance, the mannosylated antibody–enzyme fusion protein MFECP1, which selectively targets tumor cells, is cleared by the endocytic and phagocytic mannose receptor (MR) [1]. Another example is the use of mannosylated proteins in enzyme replacement therapy (ERT), such as the use of glucocerebrosidase for patients with type 1 Gaucher disease. In this case, the terminal mannose residues on the glycans are essential for targeting glucocerebrosidase to the mannose receptors of Gaucher macrophages, where most abnormal accumulation of glucocerebroside occurs [2-5].

*Yarrowia lipolytica* is a yeast expression system used for the production of heterologous proteins for therapeutic purposes [6]. It has GRAS-status (generally recognized as safe) and grows to very high cell densities on long-chain fatty acids. Moreover, the promoter for peroxisomal fatty-acyl-CoA oxidase 2 (Pox-2) can be used for inducible protein expression. In this study, we engineered the *Y. lipolytica* expression system for the production of homogeneous, human-type high-mannose glycosylated proteins, *i.e.* glycosylated with Man_8_GlcNAc_2_ or Man_5_GlcNAc_2_. We inactivated the yeast-specific Golgi α-1,6-mannosyltransferase-genes, *OCH1* and *MNN9*[7,8], which yielded a strain with very homogeneous Man_8_GlcNAc_2_ glycoproteins. To demonstrate the efficacy of this engineered strain, we overexpressed glucocerebrosidase. Further engineering involved the introduction of an ER-retained α-1,2-mannosidase. The resultant strain produced homogeneous Man_5_GlcNAc_2_ sugar structures on its glycoproteins.

## Results

### Man_8_GlcNAc_2_ N-glycan engineering by inactivating the α-1,6-mannosyltransferases *OCH1* and *MNN9*

Creating a *Y. lipolytica* strain that homogeneously attaches Man_8_GlcNAc_2_ N-glycans to recombinant glycoproteins requires engineering of the yeast N-glycosylation pathway at the Golgi level. Glycoproteins containing Man_8_GlcNAc_2_ glycans are typically elongated in the Golgi of yeast, which often results in hypermannosylation. To avoid this, we inactivated the *OCH1* gene, which encodes the Golgi α-1,6-mannosyltransferase (α-1,6ManT), which initiates hypermannosylation. Though this strategy has been successfully implemented in other yeasts [9], there have been some doubts about which of the α-1,6-mannosyltransferase-homologous genes in *Y. lipolytica* codes for this initiating transferase. It was first suggested that *Yl*Och1p (the protein most homologous to the *Saccharomyces cerevisiae* Och1p) might play only a minor role in outer-chain elongation of N-glycosylation [10], but this was later contradicted [11]. Furthermore, in the original study, more severe glycosylation defects were observed when this *MNN9* gene was inactivated than when the *OCH1* gene was inactivated, which led to the proposal that *Y. lipolytica* Mnn9p plays a major role in the Golgi N-glycosylation pathway [10]. Accordingly, we investigated the effect of knocking out *OCH1* and *MNN9* by comparing strains with single and double knock-outs of *OCH1* and *MNN9*.

**Figure 2:**
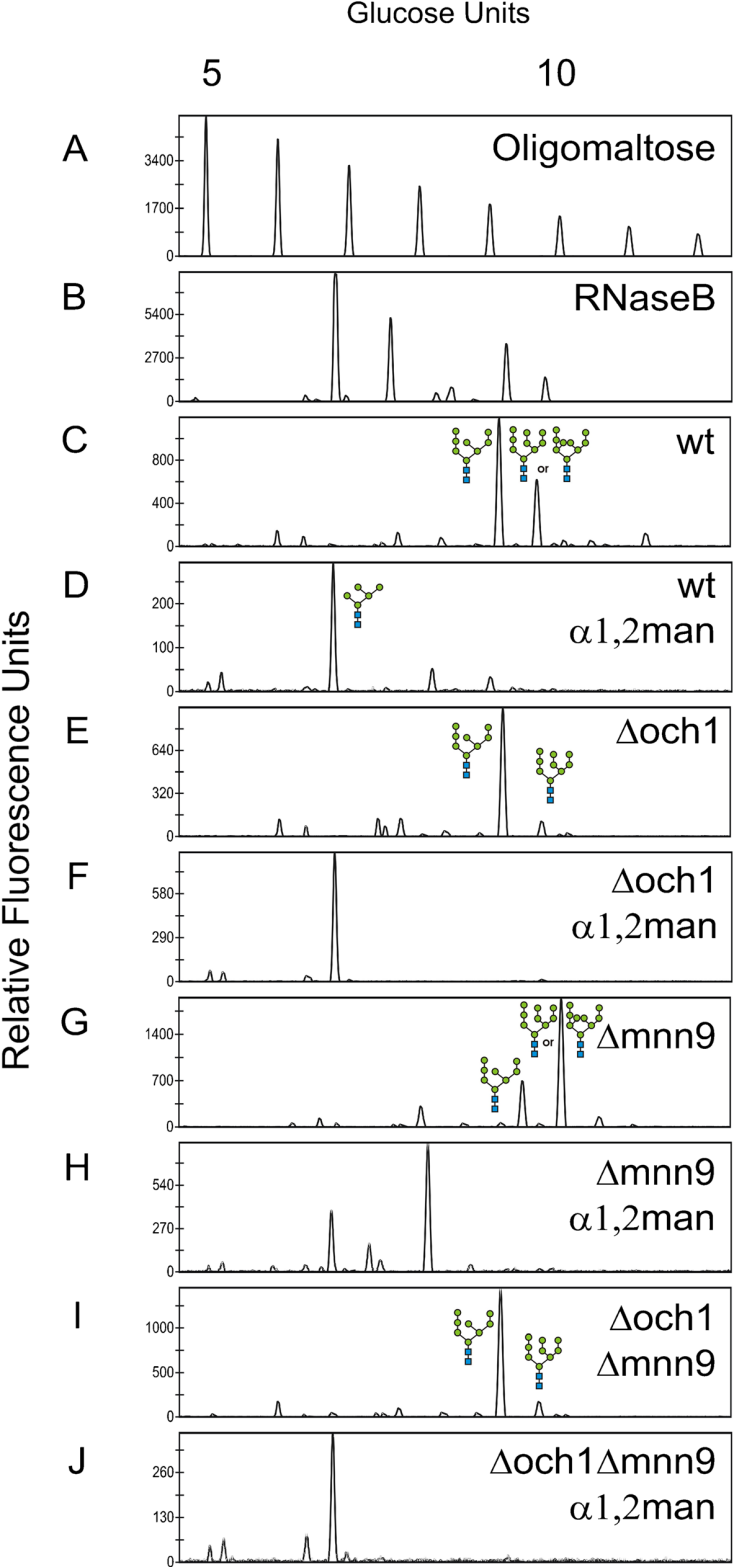
**DSA-FACE analysis of mannoprotein N-glycans derived from different *Yarrowia lipolytica *strains.** (**A**) Oligomaltose reference. (B-J) Different N-glycans. (**B**) Bovine RNaseB reference. (**C**) the MTLY60 wild type strain. (**D**) The same as panel C but treated with α-1,2-mannosidase. (**E**) The Δoch1 mutant strain. (**F**) The same as panel E but treated with α-1,2-mannosidase. (**G**) The Δmnn9 mutant strain. (**H**) The same as panel G but treated with α-1,2-mannosidase. (**I**) The Δoch1 Δmnn9 mutant strain. **(J)** The same as panel I but treated with α-1,2-mannosidase.

After inactivation of the *OCH1* gene, N-glycan analysis was performed on mannoproteins prepared from the wild-type MTLY60 and the Δoch1 mutant strain. On the glycoproteins of the Δoch1 mutant, the sugars were almost exclusively Man_8_GlcNAc_2_ with a minor fraction of Man_9_GlcNAc_2_ glycans (Figure 2E). But in the wild-type strain the oligosaccharides were more heterogeneous in size, mostly Man_8_GlcNAc_2_, Man_9_GlcNAc_2_ and larger oligosaccharides (Figure 2C). *In vitro* α-1,2-mannosidase digestion trimmed the labeled N-glycans derived from the Δoch1 mutant strain to Man_5_GlcNAc_2_ (Figure 2F), indicating that no α-1,6-mannose residue was added in the Golgi apparatus of this strain (Figure 1A, top). α-1,2-mannosidase digestion of the mannoprotein N-glycans from the wild-type strain also yielded Man_5_GlcNAc_2_. Also, a fraction of the glycans were converted to structures larger than Man_5_GlcNAc_2_ (Figure 2D), likely due to elongation with α-1,6-mannose residues.

Before inactivation of Mnn9p (another α-1,6-mannosyltransferase homolog), the Δoch1 mutant strain was first cured of its lox-flanked *URA3* marker gene. To that end, we used the Cre-lox recombination system, which is based on transformation of a transient episomal plasmid containing a cassette for expression of Cre recombinase. The *MNN9* gene was then knocked out in the *Y. lipolytica* MTLY60 wild type strain and the *URA3*-cured Δoch1 strain. Next, we analyzed the mannoprotein N-glycans derived from these strains. The Δmnn9 mutant accumulated mostly Man_9_GlcNAc_2_ N-glycans and some Man_8_GlcNAc_2_ (Figure 2G). The Man_9_GlcNAc_2_ N-glycans could not be converted to Man_5_GlcNAc_2_ by α-1,2-mannosidase digestion (Figure 2H). Moreover, glycosylation in the double mutant (Δoch1/Δmnn9) resembled that in the Δoch1 strain (Figure 2I). The N-glycans of this double mutant could be converted to Man_5_GlcNAc_2_ by α-1,2-mannosidase digestion (Figure 2J). These data are compatible with the notion that the *Y. lipolytica* Mnn9p, like its *S. cerevisiae* counterpart [12], is the α-1,6-ManT that attaches the second α-1,6-mannose to the glycan backbone after attachment of Och1p α-1,6-mannose.

**Figure 3:**
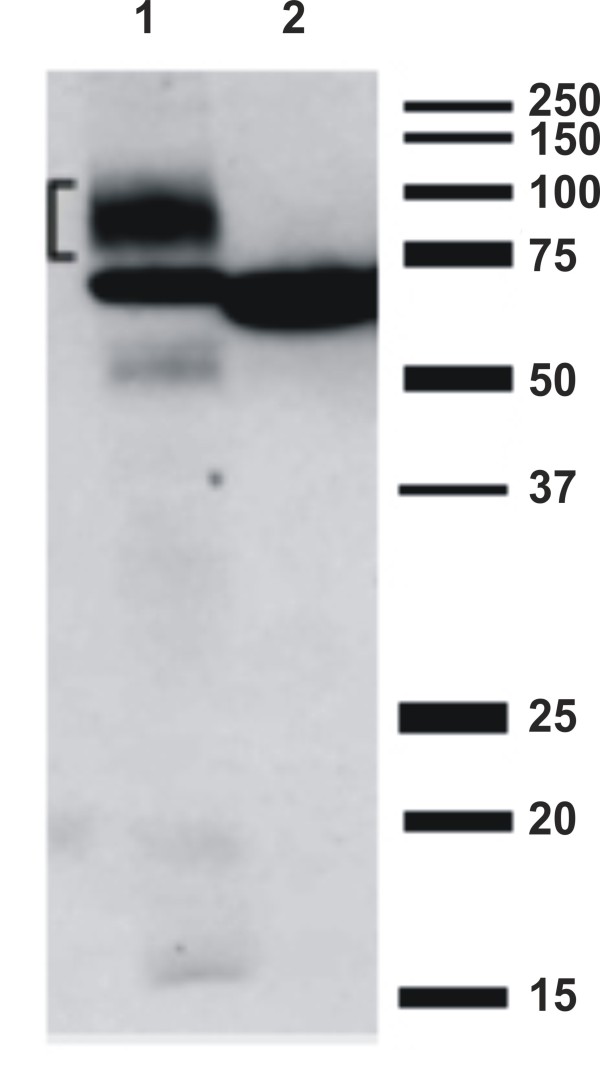
**Growth curve determination of different *Yarrowia lipolytica *strains.(A)** Growth behavior in YPD medium of wild type strain MTLY60 (♦), Δoch1 strain (■) and Δoch1 strain overexpressing a TEF promoter driven, HDEL-tagged α-1,2-mannosidase (▲). **(B)** Growth behavior in oleic acid induction medium YTO of wild type strain MTLY60 (♦), Δoch1 strain (■) and Δoch1 strain overexpressing a TEF promoter driven, HDEL-tagged α-1,2-mannosidase (▲).

The impact of glycan engineering on the growth of these engineered strains was assessed by growth curve analysis. The inactivation of the Golgi α-1,6-mannosyltransferase *Yl*och1 has no detrimental effect on the growth rate in either YPD or YTO (Figure 3 A and B).

We conclude that *YlOCH1*, as in other yeasts [7,13-16], is the initiating α-1,6-mannosyltransferase that is responsible for the yeast high mannose structures and thereby confirm the results obtained by Song et al. [11]. The Δoch1 *Y. lipolytica* strain generates an N-glycan profile that is estimated >85% Man_8_GlcNAc_2_, which is the high-mannose glycan present on proteins exported from the ER in most eukaryotes, including humans.

### Human glucocerebrosidase expression

**Figure 4:**
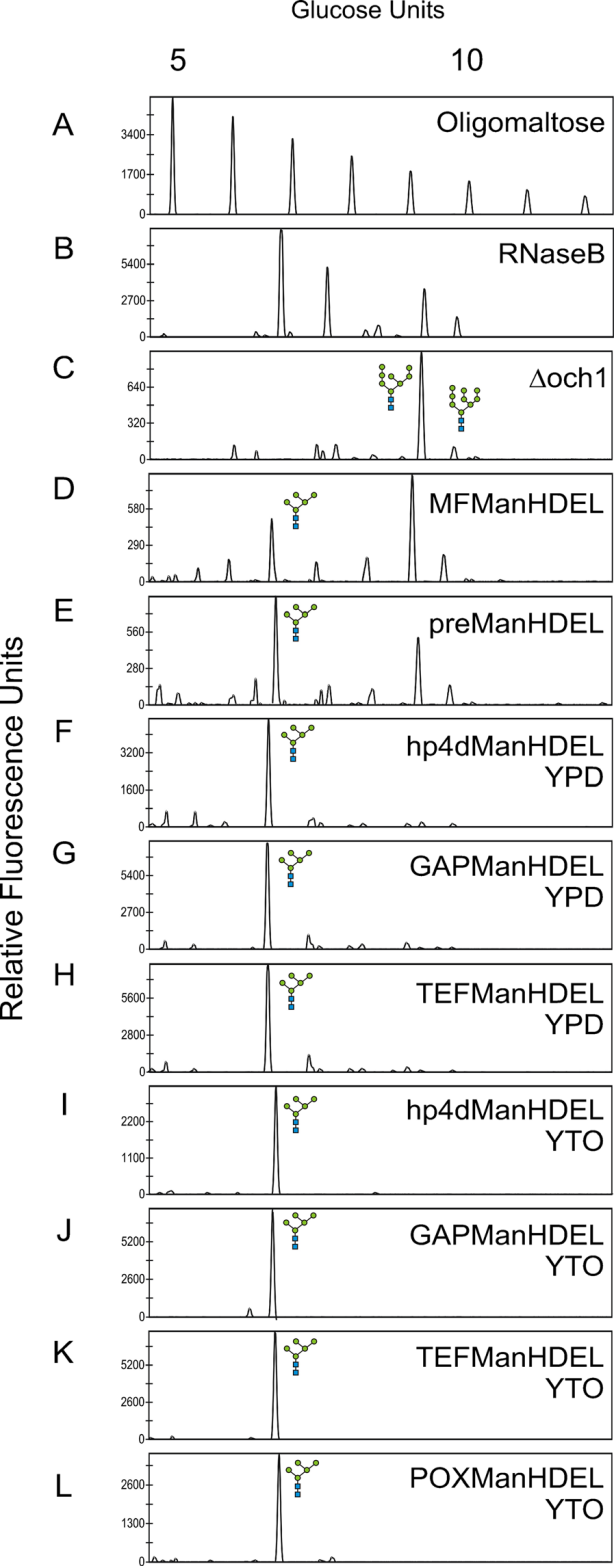
**Western blot evaluation of hypermannosylation of glucocerebrosidase before and after inactivation of *YlOCH1 *.** Expression of glucocerebrosidase by the wild-type strain (WT, MTLY60) (lane1) and by the Δoch1 mutant strain (lane2). The WT strain shows a smear, indicating hyperglycosylation, while the Δoch1 mutant strain shows a distinct band above 55 kDa, which is the MW of glucocerebrosidase without sugars.

The human glucocerebrosidase sequence [GLCM, Swiss Prot entry No. P04062] was synthesized as cDNA codon-optimized for expression in *Y. lipolytica*. The coding sequence for the mature protein (amino acids 40 to 536) was fused to the coding sequence of the LIP2pre signal sequence. This fusion construct was cloned under control of the oleic-acid-inducible POX2 promoter and transformed to the *Y. lipolytica* wild-type strain MTLY60 and the Δoch1 mutant strain. Both transformants were grown, and proteins were precipitated from the supernatant, separated by SDS-PAGE, and immunoblotted using a rat monoclonal anti- glucocerebrosidase antibody [17]. In representative immunoblots, the Δoch1 strain shows no smearing (Figure 4, lane 2), whereas WT cells show heterogeneity of the glycoprotein (Figure 4, lane 1). We conclude that inactivation of Och1p at least abolishes hypermannosylation on the test protein glucocerebrosidase.

Glucocerebrosidase was purified from a *Y. lipolytica* Δoch1 mutant strain's supernatant by a combination of cation and anion exchange chromatography and gel filtration chromatography. The purified glucocerebrosidase from the *Y. lipolytica* Δoch1 mutant strain had a specific activity on 4-Nitrophenyl-β-D-glucopyranoside of 2.5 +/− 0.8 units/mg, whereas a batch of currently used therapeutic glucocerebrosidase (Cerezyme, Genzyme Corp.) had a specific activity of 5.6 +/− 0.5 units/mg. The reason for the somewhat lower specific activity of our recombinant enzyme is most probably that no efforts have yet been done to fully optimize the fermentation, purification and formulation of this rather labile enzyme, contrary to what is the case for the clinically used Cerezyme preparations. Nevertheless, the results clearly show that the glucocerebrosidase produced in glyco-engineered *Y. lipolytica* is enzymatically active.

## Man_5_GlcNAc_2_ N-glycan engineering

### Expression of *T. reesei* α-1,2-mannosidase

To further humanize the N-glycans of *Y. lipolytica* to Man_5_GlcNAc_2_, an ER-retained Golgi-type α-1,2-mannosidase was expressed in the *URA3*-cured Δoch1 strain. The use of an HDEL-tagged *T. reesei* α-1,2-mannosidase [Genbank® Accession No. AF212153] had proven its effectiveness in hydrolyzing α-1,2-linked mannose residues *in vivo* in *Pichia pastoris*[18] as well as in *Aspergillus niger*[9]. Therefore, we chose this approach to trim down the α-1,2-linked mannoses in the Man_8_GlcNAc_2_ N-glycans of the Δoch1 strain to produce Man_5_GlcNAc_2_ N-glycans. The HDEL tag is used to target the enzyme to the ER-Golgi boundary, where its Man_8_GlcNAc_2_ substrate is formed.

**Figure 5:**
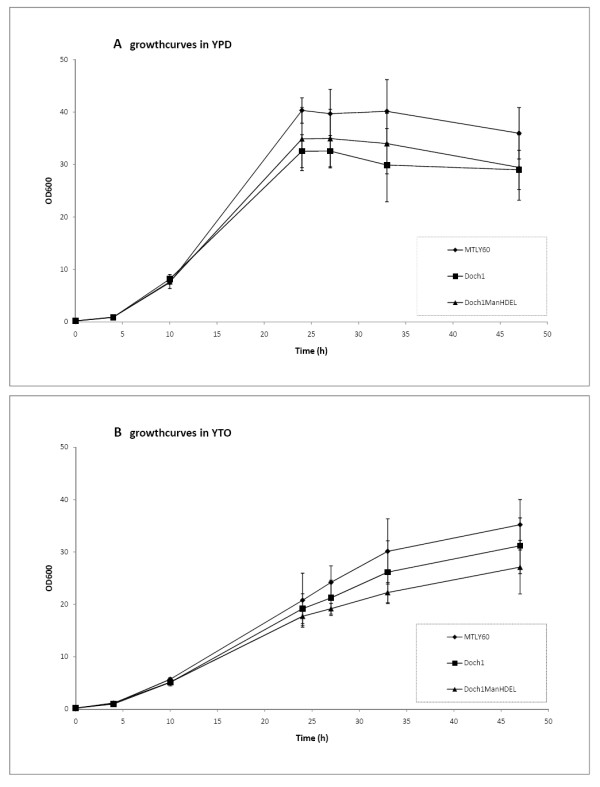
**DSA-FACE analysis of N-glycans derived from different *Yarrowia lipolytica *strains.** (**A**) Oligomaltose reference. (B-L) Different N-glycans. (**B**) Bovine RNaseB reference. (**C**) The MTLY60 wild type strain. (D-E) HDEL-tagged α-1,2-mannosidases expressed in a Δoch1 background: (**D**) the mannosidase fused to the *S. cerevisiae* α-mating factor prepro sequence and (**E**) the mannosidase fused to the *Y. lipolytica* LIP2pre. (F-L) The codon-optimized mannosidase fused to the *Y. lipolytica* LIP2pre: (**F**) under control of the hp4d promoter and grown in YPD, (**G**) under control of the GAP promoter and grown in YPD, (**H**) under control of the TEF promoter and grown in YPD, (**I**) under control of the hp4d promoter and grown in YTO, (**J**) under control of the GAP promoter and grown in YTO, (**K**) under control of the TEF promoter and grown in YTO, (**L**) under control of the POX promoter and grown in YTO.

First, we tried overexpressing the mannosidase fused to the *S. cerevisiae* α-mating factor prepro sequence under control of the constitutive hp4d promoter(pYLHmAXMFManHDEL) in the *URA3*-cured Δoch1 strain, but with very poor results. The mannoprotein N-glycan profiles show that only a minor fraction of Man_8_GlcNAc_2_ was converted to Man_5_GlcNAc_2_ (Figure 5D).

### Expression of *T. reesei* α-1,2-mannosidase using LIP2pre as secretion signal

The incomplete trimming of Man_8_GlcNAc_2_ to Man_5_GlcNAc_2_ might have been due to a non-optimal signal sequence. Therefore, we replaced the *Saccharomyces cerevisiae* α-MF prepro sequence with the secretion signal of the strongly expressed *Y. lipolytica* LIP2 gene (LIP2pre) and fused it to the coding sequence of the mannosidase, and subsequently transformed the cassette from the pYLHmAXL2preManHDEL construct to the *URA3*-cured Δoch1 strain. Some conversion of Man_8_GlcNAc_2_ to Man_5_GlcNAc_2_ occurred, but the reaction was again incomplete (Figure 5E) (Man_8_GlcNAc_2_ was present as well as the intermediate products Man_7_GlcNAc_2_ and Man_6_GlcNAc_2_). It should also be noted that much clonal variation was observed (data not shown).

### Expression of *T. reesei* α-1,2-mannosidase after codon optimization for *Y. lipolytica*

To further improve the α-1,2-mannosidase activity, we optimized codon usage and GC content of the mannosidase sequence for expression in *Y. lipolytica*. Four different promoters were used to express the codon-optimized *T. reesei* α-1,2-mannosidase in fusion with the LIP2pre signal sequence and the HDEL retrieval signal. These were (i) hp4d, a *Y. lipolytica* hybrid promoter created by Madzak et al. [19], (ii) GAP, the *Y. lipolytica* strong constitutive glyceraldehyde-3-phosphate dehydrogenase promoter, (iii) TEF1, the *Y. lipolytica* strong constitutive promoter for translation elongation factor 1, and (iv) POX2, *Y. lipolytica* peroxisomal fatty-acyl-CoA oxidase2 promoter. All four plasmids (pYLHUXL2preManHDEL, pYLGUXL2preManHDEL, pYLPUXL2preManHDEL and pYLTUXL2preManHDEL) were transformed to the *Y. lipolytica URA3*-cured Δoch1 mutant strain. The resulting strains were grown in YPD or YTO (for POX-driven expression). All constructs resulted in full conversion of cell wall mannoprotein N-glycans to Man_5_GlcNAc_2_ (Figure 5, panels F-H). To evaluate whether this would still be true in protein production conditions, all strains were analyzed after growth in the presence of oleic acid for 72 h. Again full conversion was observed (Figures 5, Panels I-L). As depicted in Figure 3, the introduction of the ER retained α-1,2-mannosidase does not severely impede growth of the engineered *Y. lipolytica* strain in either YPD or oleic acid induction medium (YTO). At the stationary phase, however, the optical density reached is lower, although not statistically significant, for the engineered than for wild type strain; a phenomenon that is seen in other glyco-engineered yeast types [9,15].

We conclude that using a signal sequence native to *Y. lipolytica* and optimizing codon usage and GC content can greatly improve the function of an enzyme expressed heterologously in *Y. lipolytica*.

## Discussion

We engineered the *Y. lipolytica* expression system to produce glycosylated proteins with homogeneous human-type N-glycans with terminal mannose, *i.e.* Man_8_GlcNAc_2_ or Man_5_GlcNAc_2_. To this end, we inactivated the *Y. lipolytica* genes that are homologous to the α-1,6-mannosyltransferase-coding sequences *OCH1* and *MNN9*[7,8]. Analysis of the N-glycan profile of cell wall mannoproteins showed that only the *YlOCH1* knock-out strain accumulated the Man_8_GlcNAc_2_ precursor glycan. These results are in agreement with a previous report [11]. Thus, though the function of Och1p in *Y. lipolytica* had been questioned [10], we conclude that the *Y. lipolytica* Och1p, like the *S. cerevisiae* α-1,6-mannosyltransferase Och1p [7,13,20,21], is critical for the extension of N-linked oligosaccharide chains, and that it is at least responsible for the addition of the first α-1,6-linked mannose to the core Man_8_GlcNAc_2_ oligosaccharide.

Moreover, we show that the hypermannosylation-related heterogeneity of glucocerebrosidase is abolished in the Δoch1 knock-out strain. Our experiments indicate that the *Y. lipolytica* Mnn9p, like the *S. cerevisiae* α-1,6-mannosyltransferase Mnn9p [12], is responsible for the attachment of the second α-1,6-mannose to the glycan backbone following Och1p activity.

After generating the Δoch1 mutant strain producing Man_8_GlcNAc_2_, we introduced an ER-retained variant of a *T. reesei* α-1,2-mannosidase. The resultant strain showed homogeneous Man_5_GlcNAc_2_ sugar structures on its glycoproteins. Several optimizations were necessary to get to this point. Comparing the best clone of both transformations, the secretion signal of the endogenous strongly expressed and secreted protein lipase2 was more effective than the often-used *S. cerevisiae* α-MFprepro signal. Moreover, we had to adapt the coding sequence, GC content (to about 50.5% GC) and codon usage to that of *Y. lipolytica*. Codon optimization has been used effectively to increase the expression of heterologous proteins in many hosts. A recent study demonstrated that codon optimization in *Y. lipolytica* increased heterologous protein production eleven-fold [22]. From our study we conclude that such optimization is also crucial for expressing the *T. reesei* α-1,2-mannosidase.

In conclusion, we report the successful generation of a *Y. lipolytica* expression platform for the production of heterologous glycoproteins that are homogenously glycosylated with either Man_8_GlcNAc_2_ or Man_5_GlcNAc_2_ N-glycans, which greatly broadens the application scope of proteins produced in this organism.

## Material and methods

### Strains, culture conditions, reagents and growth curve determination

*Escherichia coli* strains MC1061, TOP10 and DH5α were used for the amplification of recombinant plasmid DNA.

*Y. lipolytica* MTLY60 [23] and W29 [24] were used as parent strains. All yeast strains were cultured at 28°C. The media were the following: YPD (10 g/L yeast extract, 20 g/L bacto-peptone, and 20 g/L dextrose); YTO (10 g/L yeast extract, 20 g/L tryptone, 2% vol oleic acid, 0.05% vol Tween 40, 10 g/L dextrose, and 50 mM K^+^/Na^+^ phosphate buffer pH 6.8; emulsified oleic acid was added after autoclaving); MM (1.7 g/L YNB without AA and ammonium sulphate, 10 g/L glucose, 5 g/L NH_4_Cl, 50 mM K^+^/Na^+^ phosphate buffer pH 6.8, and 7.7 g/L Complex Serum-free Medium (CSM) (MP Biomedicals, Santa Ana, CA, USA), and for selection of Ura^+^ and Leu^+^ transformants, 7.7 g/L CSM –ura or CSM –leu was added instead of CSM).

To determine the growth curves of the different strains, yeast cultures started from singles colonies grown on solid YPD medium were grown overnight in liquid YPD medium. These precultures were used to inoculate 125 mL shake flasks containing 30 mL medium (YPD or YTO), to obtain an initial optical density at 600 nm (OD_600_) of 0.2. These cultures were grown at 28°C with rotation at 250 rpm and the OD_600_ was determined every few hours. All strains were examined in parallel in the same experiment.

### Standard genetic techniques

Competent *Y. lipolytica* cells were prepared as described by Barth and Gaillardin [25]. Briefly, plasmid DNA together with salmon sperm carrier DNA was added to cells pretreated with lithium acetate. PEG 4000 was then added, and after a heat shock at 39°C, the cells were plated on selective plates.

Genomic DNA was isolated using the MasterPure^TM^ Yeast DNA Purification Kit according to the manufacturer’s instructions (Epicenter Biotechnologies, Madison, Winsconsin). PCR amplification was performed in a volume of 50 μl containing 20 mM Tris–HCl pH 8.4, 50 mM KCl, different concentrations of MgCl_2_ and DMSO, 0.4 mM of dNTPs, 50 ng of template DNA, 50 pmol of primers and 2.5 units of either *Taq* or *Pfu* DNA polymerase. Cycling conditions were as follows: denaturation at 95°C for 5 min followed by a hot start at 80°C and 30 cycles of 95°C for 30 s, suitable annealing temperature for 30 s and extension at 72°C for 1.5 min, followed by 10 min of final extension at 72°C.

DNA fragments were purified from PCR reactions and gels by using NucleoSpin extract II (Macherey-Nagel, Düren, Germany).

### Vector construction

#### Knock-out of the OCH1 gene

The *OCH1* gene [GenBank® Accession No: AJ563920], complete with its promoter and its terminator, was amplified from genomic DNA of *Y. lipolytica* W29 by PCR with primers OCH1Pfw and OCH1Trv (Table 1), using *Pfu* DNA polymerase (Fermentas, Burlington, Ontario, Canada). The amplified fragment was cloned in pCR-BluntII-TOPO (Invitrogen, Carlsbad, CA, USA). The promoter (P) and terminator (T) regions were amplified from this plasmid using, respectively, primers OCH1Pfw and OCH1Prv and primers OCH1Tfw and OCH1Trv (Table 1). Because of overlapping primer sequences containing an I-*Sce*I restriction site, the two fragments could be linked by PCR with the OCH1Pfw and the OCH1Trv primers. This co-amplicon was subcloned in a pCR-BluntII-TOPO vector and Sanger-sequenced. The *URA3* selection marker flanked by *lox* sites and derived from the pKS-LPR-URA3 plasmid [26] was inserted in the introduced I-*Sce*I site between P and T to produce pYlOCH1-PUT. The desired fragment was PCR amplified with the OCH1Pfw and OCH1Trv primers and transformed to the *Y. lipolytica* MTLY60 wild type strain. Two out of twenty URA prototrophic clones found by PCR on gDNA to be Δoch1 were confirmed by Southern blot.

**Table 1:** PCR primers and oligonucleotides

Primer name	Sequence (5’… 3’)	Restriction site
OCH1Pfw	TCGCTATCACGTCTCTAGC	
OCH1Prv	CTAGGGATAACAGGGTAATGGTGTGACGAAGTATCGAG	I*-Sce*I
OCH1Tfw	CATTACCCTGTTATCCCTAGCGAGATCATGGACTGG	I*-Sce*I
OCH1Trv	ACTCTGTATACTTGTATGTATGTACTG	
MNN9Pfw	TGAGCGGCCGCTTTTCTACTTCAGAGCTGGAG	*Not*I
MNN9Prv	TAGGGATAACAGGGTAATCTTGAATGAGTATGTGTCGTG	I-*Sce*I
MNN9Tfw	ATTACCCTGTTATCCCTAGAAGGAGATGTAGCGTAAG	I-*Sce*I
MNN9Trv	GGCTTAATTAATTGGTAGTGATATAATGTAACGC	*Pac*I
LIP2prefw	GATCCATGAAGCTTTCCACCATCCTCTTCACAGCCTGCGCTACCCTGGCCGCGGTAC	
LIP2prerv	CTAGGTACCGCGGCCAGGGTAGCGCAGGCTGTGAAGAGGATGGTGGAAAGCTTCATG	
ManHDELfw	GGCAGCGCTACAAAACGTGGATCTCCCAAC	*Eco47*III
ManHDELrv	GGCCCTAGGTTACAACTCGTCGTGAGCAAG	*Avr*II

#### Knock-out of the MNN9 gene

The promoter fragment (P) of the *MNN9* gene [GenBank® Accession No: AF441127] was amplified from genomic DNA of the *Y. lipolytica* MTLY60 strain by PCR using *Taq* polymerase and the MNN9Pfw and MNN9Prv primers (Table 1). The terminator fragment (T) of the *MNN9* gene was amplified from the same DNA using primers MNN9Tfw and MNN9Trv (Table 1) and *Taq* polymerase. Again, overlap PCR with the P-forward primer and the T-reverse primer was used to link the two fragments. This co-amplicon was subcloned *Not*I–*Pac*I in pCR-II-TOPO-TA and Sanger-sequenced. The *URA3* selection marker flanked by *lox* sites and derived from pKS-LPR-URA3 was inserted in the introduced I-*Sce*I site between P and T to produce pYlMNN9-PUT. The desired fragment was excised by *Not*I–*Pac*I double digestion and transformed to the *Y. lipolytica* MTLY60 wild type strain and the *URA3*-cured Δoch1 strain. Several URA prototrophic clones were screened by PCR on gDNA to confirm correct integration of the construct.

#### Expression of the trichoderma reesei α-1,2-mannosidase HDEL

Plasmid pYLHmAXMFManHDEL was constructed to express the *Trichoderma reesei* α-1,2-mannosidase [Genbank® Accession No. AF212153] fused to the *Saccharomyces cerevisiae* prepro mating factor, tagged with a C-terminal HDEL sequence [27] and under control of the constitutive hp4d promoter [19]. The expression cassette was transformed into the *Y. lipolytica* Δoch1 strain after digestion of the plasmid with *Not*I, followed by isolation of the desired fragment using agarose-gel electrophoresis.

In another construct (pYLHUdL2preManHDEL), the *S. cerevisiae* secretion signal was replaced with the secretion signal of the *Y. lipolytica LIP2* gene (LIP2pre). The LIP2pre sequence was made by hybridizing the synthetic oligonucleotides LIP2prefw and LIP2prerv and cloning the DNA between the *Bam*HI and *Avr*II sites of pYLHmA (pINA1291) [28] to produce pYLHUdL2pre. The ManHDEL coding sequence was PCR amplified from pGAPZMFManHDEL [18] using primers ManHDELfw and ManHDELrv and cloned *Eco47*III–*Avr*II in pYLHUdL2pre, yielding pYLHUdL2preManHDEL. Next, the defective *URA3d4* selection marker cassette was, via *Mlu*I-*Avr*II digestion, replaced with the non-defective one from pYLHmAX. pYLHmAX is pYLHmA with the defective *URA3d4* selection marker removed via *Nhe*I/*Stu*I and exchanged with the *URA3* selection maker from pKS-LPR-URA3 [26] via *Kpn*I,T4 polymerase/*Avr*II. The expression cassette (with LIP2preManHDEL under control of the constitutive promoter hp4d) from the resultant pYLHmAXL2preManHDEL was transformed to the *Y. lipolytica URA3*-cured Δoch1 strain after digestion of the plasmid with *Not*I and isolation of the fragment containing the ManHDEL expression cassette.

In another construct, the *T. reesei* α-1,2 mannosidase sequence was codon-optimized for expression in *Y. lipolytica* (Geneart AG, Regensburg, Germany) and fused to the LIP2pre signal sequence. This fusion construct was expressed under control of four different promoters: (i) hp4d, a *Y. lipolytica* hybrid promoter created by Madzak et al. [19], (ii) GAP, the *Y. lipolytica* strong constitutive glyceraldehyde-3-phosphate dehydrogenase promoter, (iii) TEF1, the *Y. lipolytica* strong constitutive translation elongation factor 1 promoter, and (iv) POX2, the *Y. lipolytica* peroxisomal fatty-acyl-CoA oxidase2 promoter. (i) The mannosidase was cloned in pYLHUXL2pre, which is pYLHmAX with the LIP2pre sequence inserted via *Bam*HI-*Nco*I digestion, to produce the final pYLHUXL2preManHDEL. (ii) pYLGmAX was constructed by exchanging the hp4d promoter of pYLHmAX with the GAPPfw-GAPPrv PCR amplified GAP promoter via *Cla*I-*Bam*HI digestion. The mannosidase was then cloned in pYLGUXL2pre, which is pYLGmAX with the LIP2pre sequence inserted via *Bam*HI-*Nco*I digestion, to produce the final pYLGUXL2preManHDEL. (iii) pYLTmAX was constructed by exchanging the hp4d promoter of pYLHmAX with the TEF promoter of pYLTsA (pINA3313, a gift from J.M. Nicaud, INRA). The mannosidase was then cloned in pYLTUXL2pre, which is pYLTmAX with the LIP2pre sequence inserted via *Bam*HI-*Nco*I digestion, to produce the final pYLTUXL2preManHDEL. (iv) pYLPmAX was constructed by exchanging the hp4d promoter of pYLHmAX with the POX promoter of JMP62 (a gift from J.M. Nicaud, INRA). The mannosidase was then cloned in pYLPUXL2pre, which is pYLPmAX with the LIP2pre sequence inserted via *Bam*HI-*Nco*I digestion, to produce the final pYLPUXL2preManHDEL. All four plasmids were transformed to the *Y. lipolytica URA3*-cured Δoch1 strain after digesting the plasmid with *Not*I and isolating the fragment containing the ManHDEL expression cassette.

#### Selection marker rescue

For selection of the transformants, we made use of the Cre-lox recombination system, in which the marker is flanked by loxP and loxR sites to facilitate efficient marker rescue by transient overexpression of Cre recombinase. For overexpression of Cre recombinase, we used pRRQ2 (a gift from of J.M. Nicaud, INRA) [26], in which the enzyme open reading frame is under control of the hp4d promoter, and which carries the *LEU2* resistance gene. After transformation, strains were grown for 48 h in selective drop-out medium (MM with CSM –leu) and then tested for URA3 negative clones in which recombination occurred using a replica technique with selective plates (CSM –ura and CSM –leu). The *URA3* gene was removed from two Δoch1 clones, and screening was done by Southern blot analysis and PCR on gDNA using primers OCH1Pfw and OCH1Trv.

#### Expression of glucocerebrosidase

The human glucocerebrosidase [GLCM, Swiss Prot entry No. P04062, without the human signal sequence: PRO_0000012177] was chemically synthesized as a codon-optimized cDNA for expression in *Y. lipolytica*. The coding sequence for the mature protein was fused to the coding sequence of the LIP2pre signal sequence and placed under control of the oleic acid inducible POX2 promoter. This was done by cloning it *Eco*47III–*Avr*II in pYLPUXL2pre digested with *Sac*II, blunted with T4 DNA polymerase and then digested with *Avr*II. The resulting plasmid, pYLPUXL2preGLCM, was digested with *Not*I before use.

### Standard protein techniques

Protein expression of glucocerebrosidase controlled by the POX promoter was based on induction with oleic acid. YPD (5 mL) inoculated with precultures started from single colonies grown on solid YPD medium was incubated at 28°C for 16 h with rotation at 180 rpm. Sufficient preculture was transferred to a 250 mL shake flask containing 25 mL of YTO medium to obtain a final OD_600_ of 0.2 and incubated at 28°C with rotation at 180 rpm. After 72 h of induction, 800 μL samples were taken. Culture supernatants were precipitated using an acetone-deoxycholate-trichloroacetic acid (DOC/TCA) mixture. Glucocerebrosidase expression was analyzed by SDS-PAGE and western blot. A rat monoclonal anti-glucocerebrosidase antibody was used according to Alessandrini et al. [17].

Purification of glucocerebrosidase from the supernatant of a *Yarrowia* Δoch1 strain was performed as follows: first the medium was filtrated through a 0.22 μm filter and then diafiltrated to a buffer at pH 6.0 containing 20 mM sodium acetate, 20 mM bis-trispropane and 0.1% CHAPS. This diafiltrate was passed over a Q-Sepharose column and the flow through, containing glucocerebrosidase, was collected. Upon adjusting the pH to 5, glucocerebrosidase was purified over an S-Source omnifit column of 10 mL with an elution buffer containing 20 mM sodium acetate, 0.1% CHAPS, 1 M sodium chloride. Next, the enzyme was polished by Superdex 75 (HR10 x30) gel filtration chromatography with a buffer containing 20 mM trisodium citrate, 20 mM disodium hydrogen citrate, 0.01% (v/v) 0.15 M polysorbate 80 at pH 6). Remaining LPS after the already performed purification steps is removed by an extra injection of the final sample on a HiTrap Q column of 1 mL using the same buffer. The final concentration of glucocerebrosidase was approx. 1 mg/mL. Specific activity of the purified glucocerebrosidase was assessed with a hydrolysis assay for 4-Nitrophenyl β-D-glucopyranoside (Sigma) in a microtitre plate format. One enzyme unit is defined as the amount of enzyme that catalyses the hydrolysis of 1 μmol of 4-Nitrophenyl-β-D-glucopyranoside per minute at 37°C. Briefly, the purified enzyme samples as well as the clinically used Cerezyme positive control (Genzyme) were diluted in 50 mM sodium citrate buffer, pH 5.0. 40 μL of such samples were added to 160 μL of a 1 mM substrate solution in the same buffer and incubated for 1 hour at 37°C. Then the reaction was quenched by addition of 100 μL of 10% (w/v) sodium carbonate buffer (pH 12), which also ionizes the 4-nitrophenol for absorbance measurement at 405 nm. Upon blank substraction, the amount of product formation was calculated from a standard curve of a dilution series of 4-nitrophenol (Sigma) treated in the same way.

### Preparation of mannoproteins, N-glycan analysis and exoglycosidase digests

Mannoproteins were prepared according to Ballou et al. [29]. In brief, yeast strains were inoculated and grown overnight in 10 mL YPD medium in 50 mL falcon tubes at 28°C with rotation at 250 rpm. The cells were pelleted at 4000 rpm in a cooled Eppendorf 5810R centrifuge. They were first washed with 2 mL of 0.9% NaCl solution and then twice with 2 mL of water, and then resuspended in 1.5 mL of 0.02 M sodium citrate pH 7. After autoclaving for 90 min at 121°C, they were vortexed and the cellular debris was removed by centrifugation. The mannoproteins in the supernatant were precipitated overnight with 4 volumes of methanol at 4°C on a rotating wheel. After centrifugation, the pellets were allowed to dry and dissolved in 50 μL of water.

The whole 50 μL of the cell wall extract was used to prepare N-glycans labeled with 8-aminopyrene-1.3.6-trisulphonic acid (APTS) according to an established method involving the blotting of the proteins to PVDF [30]. Subsequently, fluorophore-assisted carbohydrate electrophoresis (FACE) was performed with an ABI 3130 DNA sequencer.

APTS-labeled glycans were treated with α-1,2-mannosidase overnight at 37°C in 50 mM ammonium acetate (pH 5.0).

## Competing interests

N C., K.D.P. and W.V. are inventors on a patent application claiming some of the inventions in this publication. W.V. and A.V. are employees of Oxyrane Belgium N.V. which has obtained the exploitation rights of this patent application.

## Authors’ contributions

KDP: drafting the article; intellectual contribution. WV: acquisition and analysis of data; intellectual contribution. ID: acquisition of data. AV: acquisition of data. AVH: acquisition of data. NC: initiated and managed the project, contributed to manuscript drafting. All authors read and approved the final manuscript.
